# Three-Dimensionally Porous Li-Ion and Li-S Battery Cathodes: A Mini Review for Preparation Methods and Energy-Storage Performance

**DOI:** 10.3390/nano9030441

**Published:** 2019-03-15

**Authors:** Jinyun Liu, Jiawei Long, Sen Du, Bai Sun, Shuguang Zhu, Jinjin Li

**Affiliations:** 1 Key Laboratory of Functional Molecular Solids, Ministry of Education, College of Chemistry and Materials Science, Anhui Normal University, Wuhu 241002, China; jwlong@ahnu.edu.cn; 2 Key Laboratory for Thin Film and Micro Fabrication, Ministry of Education, Department of Micro/Nano Electronics, Shanghai Jiao Tong University, Shanghai 200240, China; du_sen@sjtu.edu.cn; 3 Department of Environmental Engineering, College of Environment and Energy Engineering, Anhui Jianzhu University, Hefei 230601, China; bsun@mail.ustc.edu.cn (B.S.); zhushuguang@ahjzu.edu.cn (S.Z.)

**Keywords:** secondary battery, nanostructure, porosity, capacity, stability

## Abstract

Among many types of batteries, Li-ion and Li-S batteries have been of great interest because of their high energy density, low self-discharge, and non-memory effect, among other aspects. Emerging applications require batteries with higher performance factors, such as capacity and cycling life, which have motivated many research efforts on constructing high-performance anode and cathode materials. Herein, recent research about cathode materials are particularly focused on. Low electron and ion conductivities and poor electrode stability remain great challenges. Three-dimensional (3D) porous nanostructures commonly exhibit unique properties, such as good Li^+^ ion diffusion, short electron transfer pathway, robust mechanical strength, and sufficient space for volume change accommodation during charge/discharge, which make them promising for high-performance cathodes in batteries. A comprehensive summary about some cutting-edge investigations of Li-ion and Li-S battery cathodes is presented. As demonstrative examples, LiCoO_2_, LiMn_2_O_4_, LiFePO_4_, V_2_O_5_, and LiNi_1−x−y_Co_x_Mn_y_O_2_ in pristine and modified forms with a 3D porous structure for Li-ion batteries are introduced, with a particular focus on their preparation methods. Additionally, S loaded on 3D scaffolds for Li-S batteries is discussed. In addition, the main challenges and potential directions for next generation cathodes have been indicated, which would be beneficial to researchers and engineers developing high-performance electrodes for advanced secondary batteries.

## 1. Introduction

**Figure 1:**
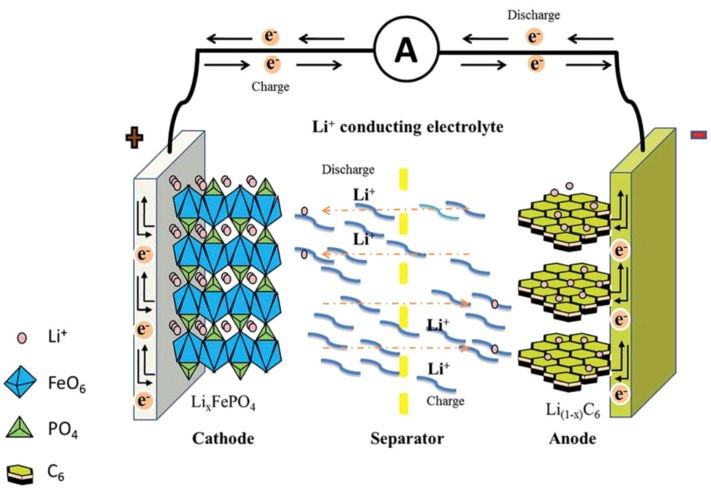
Schematic illustration for the working mechanism of a typical LiFePO_4_ cathode-based Li-ion battery. Reproduced with permission from [12]. The Royal Society of Chemistry, 2014.

**Figure 2:**
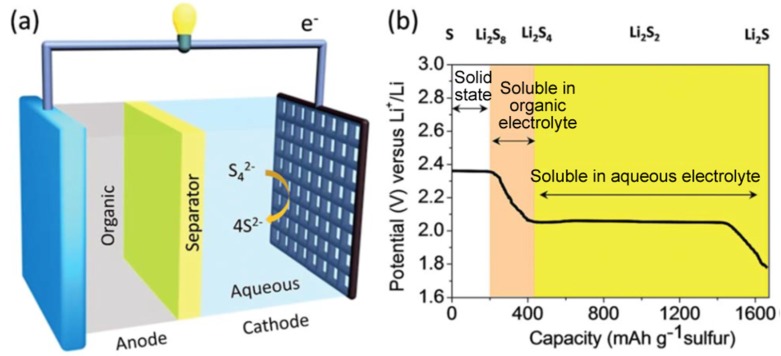
(**a**) Illustration of a Li-S battery and the (**b**) electrochemical species formed during lithiation. Reproduced with permission from [14]. The Royal Society of Chemistry, 2015.

Rechargeable batteries are widely used in emergency power backup, electric vehicles, solar power storage, portable equipment, and wearable electronics [1,2]. Among them, Li-ion and Li-S batteries have competitive advantages to the others [3,4,5,6,7,8,9]. The principle of Li-ion batteries is the insertion and extraction of Li ions in the electrodes during charge and discharge [10,11,12,13], as illustrated in Figure 1. The electricity outside the battery is carried by electrons around the external circuit in the opposite direction to Li ions. For Li-S batteries, as displayed in Figure 2, S_8_ reacts with Li ions to form high-order lithium polysulfides Li_2_S_x_ (4 < x < 8), and then lower-order lithium polysulfides Li_2_S_x_ (2 < x < 4) are formed as the Li ions insert continuously [14,15,16].

Currently, cathode materials are one of the key points for the development of high-performance Li-ion batteries and Li-S batteries [17,18]. Many cathode materials, almost with porous characteristics, have the advantages of enlarged surface area, increased specific capacity, and are able to overcome volume variation. Among various structures, three-dimensional (3D) porous structures are considered ideal and promising. 

It is necessary to improve the kinetics of the extraction/insertion process of Li ion in Li-ion batteries and the reaction between Li ions and S in Li-S batteries, which can be facilitated by constructing a 3D porous morphology. In addition, 3D porous structures have several void spaces, which are able to improve the contact with electrolytes. However, a part of the electrolyte is needed to wet the active materials during the charge/discharge process, therefore the 3D structure would consume more electrolytes [19,20,21]. In addition, the reduced material loading within the 3D electrode also needs to be improved for practical applications. Both the advantages and drawbacks of the 3D porous electrodes make it an attractive field for intensive study. Herein, we focus on the 3D porous cathode materials used in both Li-ion and Li-S batteries and their preparation methods.

## 2. Challenges of Li-Ion and Li-S Battery Cathodes

Performances of Li-ion batteries are partly dependent on the intrinsic property of the materials used in the cathodes. For cathode materials, high specific capacity, high potential, large ionic and electronic transport rate, and long life are preferred. Nowadays, most of the cathode materials are Li-based intercalation compounds. The available compounds, such as LiNiO_2_, LiCoO_2_, and LiMnO_2_, are investigated for cathode applications. To achieve a high energy-density, some compounds composed of multiple metal atoms, such as LiNi_1/3_Co_1/3_Mn_1/3_O_2_ (NCM), are expected to replace part of the single metal compounds [22]. Compounds with MO_6_ octahedra (where M is Fe, Mg, or V) and XO_4_ n– tetrahedral anions (where X is Si or P) are of interest because these structures can tune transition metal redox potentials, such as Fe^3+^/Fe^2+^. However, these kinds of compounds have a common and fundamental problem in that the conductivity is poor, which reduces the electrochemical performance.

Compared to Li-ion batteries, Li-S batteries have a much higher theoretical energy density (2600 Wh kg^−1^) and better capacity (1672 mAh g^−1^) [23]. However, severe issues of sulfur need to be addressed. At first, elemental sulfur is a natural insulator (5 × 10^−30^ S cm^−1^), which absolutely restricts the movements of electrons. The long-chain lithium polysulfide formed during the discharge process tends to dissolve in the organic electrolytes. When these polysulfides diffuse to the anode through the electrolytes, and react with the lithium anode, the reactions are named as shuttle mechanisms. During the transition between long-chain and short-chain lithium polysulfides, partial electrical energy is consumed and this unexpected phenomenon restricts the electrical efficiency. Meanwhile, an unstable film will be generated because of such uncontrolled reactions. As a result, the instability of solid electrolyte interface (SEI) film has a negative effect on the cycling stability of Li-S batteries. It is mentioned that the mass density of sulfur (2.03 g cm^−3^) is about 20% larger than that of Li_2_S (1.67 g cm^−3^), which causes the non-ignorable shrink and expansion during charge and discharge. Last but not least, the morphology of the lithium electrode is changeable due to the deposition of lithium ions from electrolytes. Dendrites of lithium growing on the surface of the electrode should be removed to ensure battery safety.

## 3. Methods for Making 3D Porous Li-Ion Battery Cathodes

### 3.1. Hydrothermal Synthesis

Hydrothermal methods include the various techniques for crystallizing materials from high-temperature aqueous solutions at high vapor pressures. Compared with several other types of crystal growth, the hydrothermal approach is able to create crystalline phases that are not stable at the melting point. In addition, materials that have a high vapor pressure near their melting points can be grown by the hydrothermal method. These advantages make the hydrothermal method suitable for constructing many 3D electrodes for batteries. This section will mainly focus on the hydrothermal methods.

The conventional LiFePO_4_ cathode has a poor electronic conductivity (~10^−9^ S cm^−1^) and slow lithium ion diffusion, which restricts its wide application [24]. Several methods have been performed to enhance the electric conductivity and the rate of ion transport, including reducing the size of LiFePO_4_ to the nanometer scale, coating particles with conductive materials, or expanding surface areas through structure manufacture. A 3D current collector of carbon layer coated LiFePO_4_ was constructed, in order to improve the high-rate discharge capacity compared to conventional cells using the foil current collector. Furthermore, the carbon layer on LiFePO_4_ nanoparticles with a diameter between 70 nm and 100 nm protects these particles and also contributes to conductivity enhancement [25]. In addition, the porous substrates made of nickel and chromium not only improve electric conductivity but also produce a large surface area. 

Du et al. reported a facile in situ one-pot hydrothermal method for preparing a conductive graphene (G)/LiFePO_4_ composite [26]. When LiFePO_4_ nanoparticles load on the 3D graphene network, rapid electronic transmission and a short lithium ion transfer pathway can be achieved. The 3D G/LiFePO_4_ composite exhibited a high capacity of 160 mAh g^−1^ at 0.2 C (94.12% of its theoretical capacity of 170 mAh g^−1^), a good rate performance of 115 mAh g^−1^ at 10 C (71.9% of its initial capacity), and good cycling performance of 94.2% capacity retention after 100 cycles, all of which imply their potential application in high rate Li-ion batteries.

Fu et al. [27] presented a novel hollow hierarchical structured composite of 0.5Li_2_MnO_3_·0.5LiMn_0.4_Co_0.3_Ni_0.3_O_2_ with a flower-like morphology. The presence of the internal cavity in the nanoplates, which consist of the complete flower-like composites, indicates that the hollow shell can provide channels for electrolytes and ions. The discharge capacities are 296.5, 270.6, 243.6, 207.8, and 187.4 mAh g^−1^ at rates of 0.2, 0.5, 1, 3, and 5 C, respectively. The capacity retention is over 87% after 100 cycles at 0.5 C with a capacity fading rate of 0.13% per cycle.

**Figure 3:**
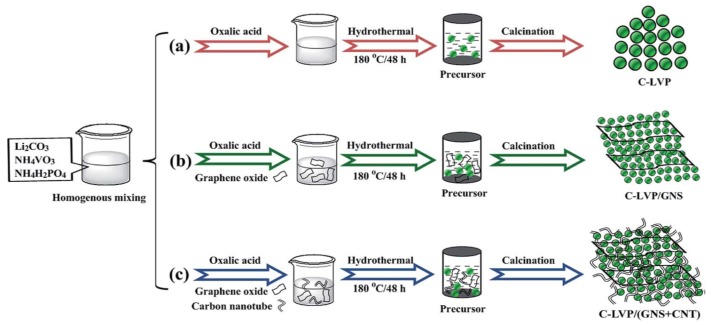
Schematic illustration of preparing nanosized Li_3_V_2_(PO_4_)_3_/C (C-LVP), carbon nanotube modified Li_3_V_2_(PO_4_)_3_/C (C-LVP/GNS), graphene nanosheet, and carbon nanotube co-modified Li_3_V_2_(PO_4_)_3_/C (C-LVP/(GNS+CNT)) composites via a hydrothermal-assisted sol-gel route. Reproduced with permission from [29]. The Royal Society of Chemistry, 2016.

**Figure 4:**
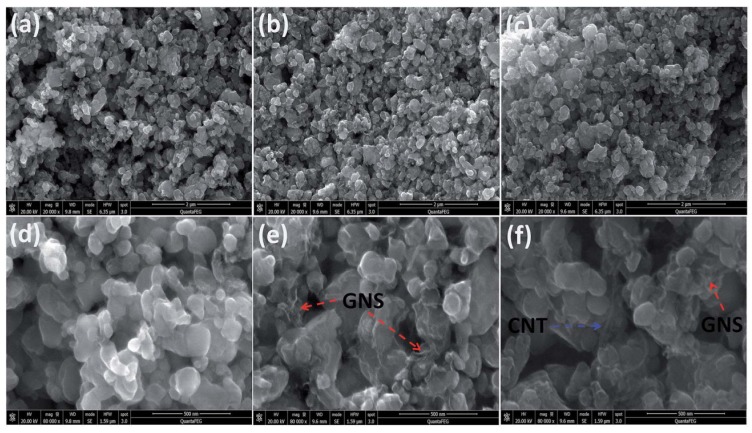
Scanning electron microscopy (SEM) images of (**a**,**d**) C-LVP, (**b**,**e**) C-LVP/GNS, and (**c**,**f**) C-LVP/(GNS+CNT). Reproduced with permission from [29]. The Royal Society of Chemistry, 2016.

Li_3_V_2_(PO_4_)_3_, with the crystal structure of monocline, is promising for the cathode material in Li-ion batteries because of its inherent characteristics of a high working potential of 4.8 V, theoretical capacity of 197 mAh g^−1^, and higher energy density of 800 Wh kg^−1^ [28]. Li_3_V_2_(PO_4_)_3_ phase consists of a 3D framework of slightly distorted VO_6_ octahedra- and PO_4_ tetrahedra-sharing oxygen vertexes, which host Li ions in relatively large interstitial sites, leading to fast ionic transport. Cui et al. prepared carbon-coated Li_3_V_2_(PO_4_)_3_ nanocrystals that were modified by graphene nanosheets and carbon nanotubes through a hydrothermal method [29], as shown in Figure 3. The graphene nanosheets and the carbon nanotubes are interconnected to form a 3D conductive network, which also support Li_3_V_2_(PO_4_)_3_ particles. When using graphene sheets, the aggregated Li_3_V_2_(PO_4_)_3_/C nanoparticles load on graphene sheets, forming a 3D layered structure. When the carbon nanotubes were also used, the interconnection between each graphene layer was improved. The carbon nanotubes enhance the structure strength and provide pathways for electron transfer. Considering the shaggy structure of the composites (Figure 4), the composite exhibits a remarkably high rate capability and long cycle stability. An initial discharge capacity of 147.5 mAh g^−1^ at 20 C at a potential range of 3.0–4.8 V is obtained. The capacity retention is 82.7% after 2000 cycles.

Vanadium pentoxide (V_2_O_5_) is a typical intercalation compound. It possesses a layered structure that can accept more than one electron and lithium ion through the reaction V_2_O_5_ + xLi^+^ + xe^−^ ↔ Li_x_V_2_O_5_. However, V_2_O_5_ has shortcomings, with a moderate electrical conductivity of 10^−4^–10^−5^ S cm^−1^ and low Li^+^ diffusion coefficient of 10^−12^ cm^2^ s^−1^ [30]. As is known, binder-free technology loads active materials on the current collector without any binders and conductive adhesives, enabling a simplified electrode for batteries. Many free-standing 3D porous V_2_O_5_ electrodes have been reported. For example, Gao et al. reported a one-step hydrothermal method to confine vanadium pentoxide particles in 3D N-doped graphene as a free-standing cathode [31]. The good conductivity and elastic properties of N-doped graphene are utilized efficiently. The composite cathode shows a high capacity of 283 mAh g^−1^ at 100 mA g^−1^ (96.3% of its theoretical capacity of 294 mAh g^−1^) and a good rate performance of 134 mA g^−1^ at 1 A g^−1^ (78% of its initial capacity).

**Figure 5:**
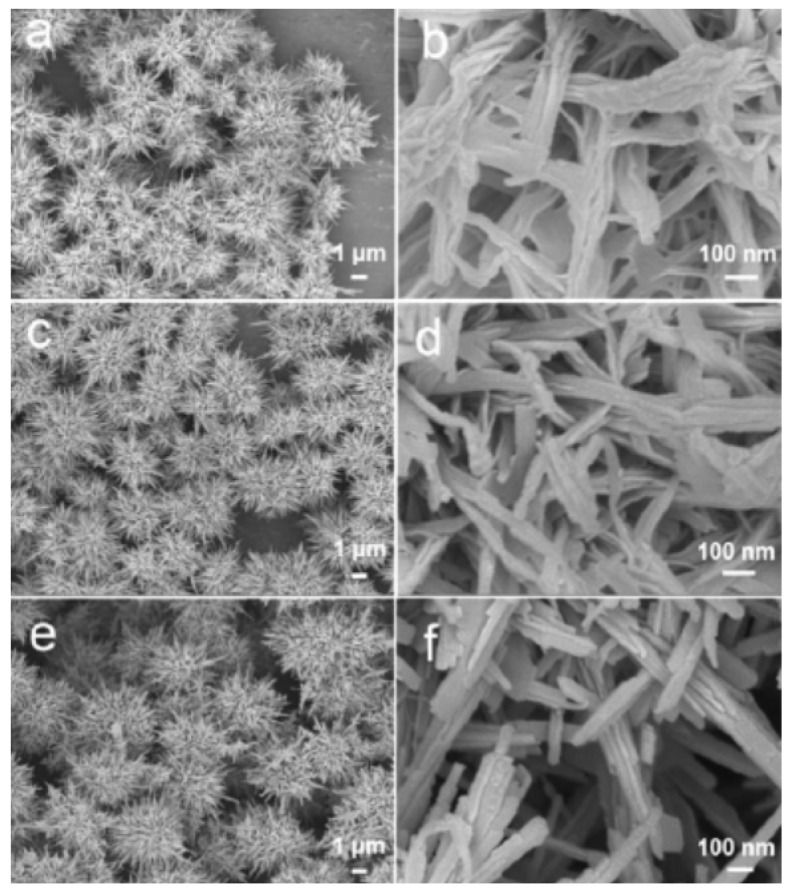
SEM images of the 3D vanadium oxide obtained after different solvothermal reaction time: (**a**,**b**) VO-0.5 h, (**c**,**d**) VO-2 h, and (**e**,**f**) VO-24 h. Reproduced with permission from [32]. American Chemical Society, 2012.

In addition, 3D hierarchical nanostructures are widely applied in electrodes because such structures suppress the agglomeration and improve the diffusion of lithium ions. Pan et al. found that the precursor of vanadyl oxalate (VOC_2_O_4_) at different concentrations impacts on the morphology of the products [32]. As seen in Figure 5, they reported a facile solvothermal method to produce hierarchical nano and microstructures of vanadium oxide by adjusting the concentration of VOC_2_O_4_ and the duration time. Urchin-like VO_2_ microflowers were first synthesized without surfactants through self-assembly, which were followed by calcination to transform VO_2_ into urchin-like V_2_O_5_ microstructures. The V_2_O_5_ microstructures were composed of nanobelts and nanorods, in which a highly porous texture was confirmed. The obtained V_2_O_5_ had a specific area of 33.64 m^2^ g^−1^, which is ascribed to the 3D hierarchical porous structure and the large surface area of the building blocks. In addition, when evaluated as a cathode material, the V_2_O_5_ cathode delivers a high specific discharge capacity of 267 mAh g^−1^ at a current density of 300 mAh g^−1^.

Guo et al. reported a simple hydrothermal method for the synthesis of a fine V_2_O_5_-SnO_2_/CNTs composite, in which the nanosized V_2_O_5_ coating and the carbon nanotubes (CNTs) were connected through SnO_2_ nanoparticles [33]. After heat treatment, the composites were verified to be porous due to the stack of CNTs. The external surface of the CNTs was uniformly covered with SnO_2_ and V_2_O_5_, which improved the cyclic capacity and the rate capacity as a result of the excellent conductivity. The V_2_O_5_-SnO_2_/CNTs composite cathode exhibited an initial discharge capacity of 250 mAh g^−1^ from 2.05 to 4.0 V. Furthermore, a high coulombic efficiency (over 99%) for all cycles was achieved, which showed an excellent cycling stability.

**Figure 6:**
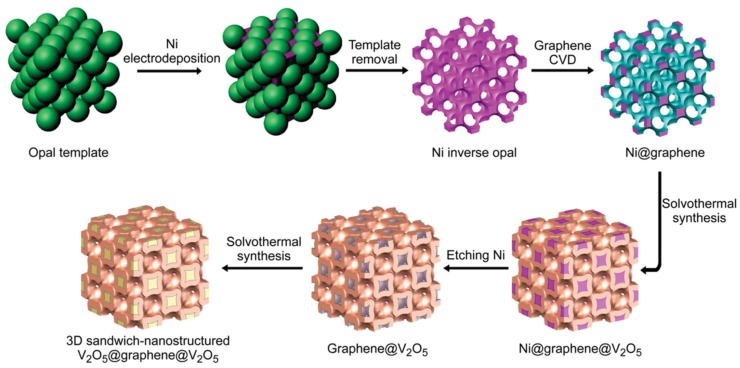
Schematic illustration for the fabrication of the 3D sandwich-structured V_2_O_5_@graphene@V_2_O_5_ cathode. Reproduced with permission from [34]. Wiley, 2016.

Our group has presented a facile method for manufacturing 3D structural electrodes by integrating active materials and graphene [34]. As displayed in Figure 6, a sandwich-like structure of V_2_O_5_@graphene@V_2_O_5_ was fabricated for the cathode based on the 3D inverse opal template. At first, a Ni inverse opal was electrodeposited on a polystyrene opal template, on which graphene was then grown via chemical vapor deposition. After a Ni etching process, a layer of V_2_O_5_ was grown onto the external surface of graphene. Then, another layer of V_2_O_5_ was grown into the inner layer of graphene, forming the 3D V_2_O_5_@graphene@V_2_O_5_ composite. The cathode provided a full electrode basis capacity of about 230 mAh g^−1^ at 5 C after 200 cycles, and 203 mAh g^−1^ after 2000 cycles, along with a Coulombic efficiency of 99.7%.

### 3.2. Sol-Gel Method

Another widely used method for preparing 3D electrodes is sol-gel. For example, Park et al. reported a sol-gel method with polystyrene beads as templates to prepare 3D LiMn_2_O_4_ thin films. A monolayer of polystyrene microspheres was first deposited on the surface of the substrates, which was the basis for manufacturing an open volume without overlaps in the perpendicular direction [35]. Subsequently, the prepared solvents containing precursor materials were dipped onto the substrates. After the calcination of deposited films, the polystyrene template was removed by the thermal decomposition. The precursor was converted to LiMn_2_O_4_. At last, a 3D spherical porous structure with an inverse-opal was formed. It was found that discharge capacity was associated with the annealing temperature, which has strong effects on the stable structure. Zhang et al. successfully encapsulated Li_3_V_2_(PO_4_)_3_ nanoparticles in amorphous carbon via a facile sol-gel method [36]. The presence of thin carbon layer and carbon network benefit Li^+^ transport and electron conductivity. Applied as the cathode, this material exhibits a capacity of 85 mAh g^−1^ at a high rate of 30 C.

Li et al. reported a sol-gel method to construct the 3D LiAlO_2_-LiMnPO_4_/C for a Li-ion battery [37]. The LiAiO_2_ template was first constructed from an anodic aluminum oxide (AAO) template. Then, LiMnPO_4_/C filled in the pores of the prepared template, and finally formed a honeycomb-like structure. When used as the cathode of a Li-ion battery, this material shows a high capability of 105 mAh g^−1^ and 98.4% retention after 100 cycles at 10 C. The electrochemical performance has been improved due to the special core-shell architectures.

Ragupathi et al. reported a sol-gel method to synthesize spherical LiCoBO_3_ for use as the cathode material of a Li-ion battery [38]. The as-prepared material presents a capacity of 98 mAh g^−1^ at 10 C after 52 cycles. The electrochemical performance of this material is superior to others derived from different methods, which may be ascribed to the formation of its spherical shape, which facilitates electron movement and Li-ion diffusion.

The sol-gel method is commonly low-cost and simple, which make it appropriate for preparing electrodes with a large yield. Particularly, their industrial manufacture always contains a sol-gel preparation.

### 3.3. Solid-Based Approcah

Despite the wet-chemical strategies introduced above, some solid-based approaches have also attracted intensive attention. Wang et al. successfully utilized a topochemical reaction route to synthesize porous LiMn_2_O_4_ spheres assembled by nanocrystalline, which took advantage of the porosity of Mn_2_O_3_ spheres obtained from the thermal decomposition of MnCO_3_ [39]. TEM and N_2_ adsorption-desorption measurements showed that the whole LiMn_2_O_4_ microspheres have an average size of about 45 nm. It was observed that the interior of LiMn_2_O_4_ spheres, which showed a good performance in terms of capacity and cycling, were filled of abundant pores. The discharge capacity is 83 mAh g^−1^ at a rate of 20 C, which may be ascribed to the porous channels that are suitable for Li ion diffusion.

**Figure 7:**
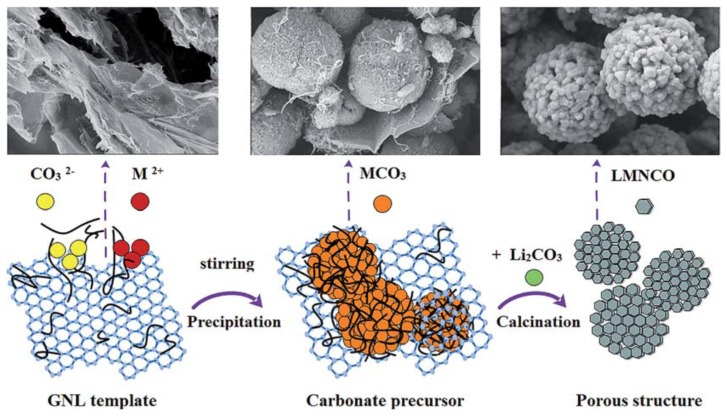
Formation mechanism of the porous graphene and carbon nanotube conductive liquid- Li_1.2_Mn_0.534_Ni_0.133_Co_0.133_O_2_ cathode. Reproduced with permission from [40]. American Chemical Society, 2015.

Huang et al. [40] used graphene and carbon nanotube conductive liquid (GNL) as a template to synthesize porous Li_1.2_Mn_0.534_Ni_0.133_Co_0.133_O_2_, as displayed in Figure 7. As for the GNL, CNTs are uniformly distributed on the layer of the graphene sheets. The manufacturing process of the composite is dependent on the preparation of Li_1.2_Mn_0.534_Ni_0.133_Co_0.133_O_2_ through a high-temperature calcination of spherical precursors of transition metal carbonate. For the porous GNL-LMNCO with the spherical particles, the secondary particles are composed of the primary particles with sizes of 150–180 nm. The spheres of the secondary particles almost possess the same size of 2.0 mm. A great number of pores can be obviously detected from the stacking of the primary particles. The porous Li-rich cathode delivers a discharge capacity of 235.5 mAh g^−1^, even after 100 cycles of charge/discharge, which suggests a high capacity retention of 86.2% compared to the initial capacity.

In one study, de Biasi et al. synthesized a trigonal colquiriite-type crystal structure LiCaFeF6 via solid-state reaction approach [41]. This material’s potential may be thanks to its safety and cycle life. When used as a cathode material, it exhibits a discharge capacity of 112 mAh g^−1^ and a maximum discharge current of 2.86 V. This was assigned to the reduction of Fe^3+^ to Fe^2+^.

Baster et al. synthesized a series of LiNi_0.6_Co_0.4−Z_Ti_Z_O_2_ (z = 0.1, 0.2, 0.3) for the cathode materials of Li-ion batteries and compared their structural, transport, and electrochemical properties [42]. Of three materials, LiNi_0.6_Co_0.3_Ti_0.1_O_2_ exhibits a discharge capacity of 100 mAh g^−1^ at 20 C.

### 3.4. Other Methods

Differing from the methods shown above, there are some other preparation approaches for making 3D cathodes for Li-ion batteries, including freeze-drying, chemical vapor deposition, vacuum filtration, and others. For instance, Doherty et al. synthesized beads of poly methyl methacrylate with different diameters as colloidal crystal templates to produce LiFePO_4_ with featured pores [43]. The use of colloidal crystal templates allowed an examination of the effects of pore size in terms of the electrochemical properties. The materials with the largest pores, around 100 nm diameter, exhibited the best discharge capacities of 160 mAh g^−1^ at 0.1 C and 115 mA h g^−1^ at 5 C, respectively.

Tu et al. synthesized the 3D porous LiFePO_4_ materials modified with the uniformly dispersed nitrogen-doped carbon nanotubes (N-CNTs) by a freeze-drying method [44]. It included three steps: rapid freezing, vacuum freeze-drying, and a heating treatment. The synthesized porous LiFePO_4_/NCNTs composite demonstrates a specific capacity of 159 mAh g^−1^ at a rate of 0.1 C, a rate performance with a capacity of 72 mAh g^−1^ at a high rate of 10 C, and a high capacity retention of 96.7% after 500 charge/discharge cycles.

Zhao et al. synthesized an orthorhombic LiMnO_2_ nanorod by in-situ carbothermal reduction [45]. This approach included two steps: preparation of MnO_2_ nanorod and carbothermal reduction to obtain LiMnO_2_. When used as a cathode in a Li-ion battery, this material exhibits outstanding electrochemical performance of 165.3 mAh g^−1^ at 0.1 C and 92.6% retention after 40 cycles. As for manganates, Duan et al. synthesized the LiMn_2_O_4_ hollow nanofibers with a porous structure by modified electrospinning techniques on the fluorine-doped tin oxide glass [46]. The cathode made of these hollow materials delivered a specific capacity of 125.9 mAh g^−1^ and a cycling performance of 105.2 mAh g^−1^ after 400 cycles at 0.1 C, which exhibited a good battery performance.

**Figure 8:**
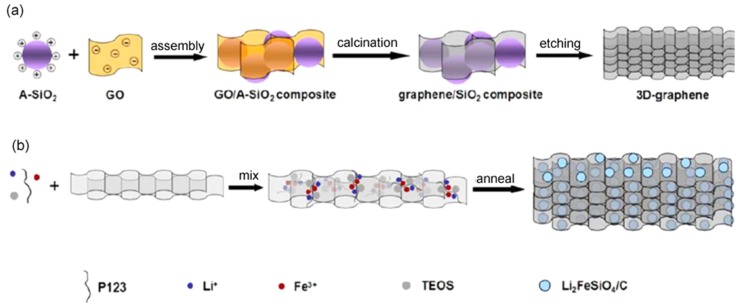
Illustrations of the synthesis procedures of (**a**) the 3D porous graphene and (**b**) the graphene/Li_2_FeSiO_4_/C composite. Reproduced with permission from [49]. American Chemical Society, 2014.

A 3D mace-like Li_3_V_2_(PO_4_)_3_/C nanowire and nanofiber membrane was prepared by using nanoparticles as a catalyst [47]. The fabrication process combines a modified electrospinning method with a hot-press treatment. The surface of the fibers is covered with nanowires that grow in situ, forming a mace-like morphology. Based on the mechanical property of the composite, the long-range-networking membrane is directly available for self-standing cathodes. The obtained cathode exhibits a good rate performance and cyclic stability in the voltage range of 3.0–4.8 V. Discharge capacities of 115.3 mAh g^−1^ and 108.6 mAh g^−1^ at 5 C and 10 C are achieved, respectively. As for the capacity retention, it is 81.4% after 500 cycles, and 78.8% after 1000 cycles, showing quite good stability. Li et al. successfully synthesized a Li_3_V_2_(PO_4_)_3_/C composite with 3D foam-core structure via a facile immersion method [48]. The uniform Li_3_V_2_(PO_4_)_3_/C bubbles were observed. Li_3_V_2_(PO_4_)_3_ nanoparticles were coated uniformly by an amorphous carbon layer. The cathode exhibits a good rate performance with a specific capacity of 100 mAh g^−1^ at 15 C in the potential range of 3.0–4.3 V, which is attributed to the 3D porous frog egg-like structure. Zhu et al. reported the synthesis of 3D microporous graphene-Li_2_FeSiO_4_/C nanocomposites [49]. As displayed in Figure 8, the 3D porous graphene framework was obtained through a templated-assembly method. During the preparation process, the surface grafted and positively charged SiO_2_ spheres were assembled with negatively charged graphene oxide (GO) sheets by electrostatic interactions. The SiO_2_ spheres were then removed, forming a 3D porous graphene framework. Finally, a Li_2_FeSiO_4_ precursor gel was coated and annealed to get the 3D G/Li_2_FeSiO_4_/C composite. These composite have the advantage of the porosity of a 3D structure and the conductivity of graphene, with the discharge capacities reaching 255 mAh g^−1^ at 1 C. 

In addition, a flexible, free standing, and light-weight LiMnTiO_4_/MWCNT was synthesized by vacuum filtration method [50]. The prepared material possesses a 3D structure, where CNT networks are well wrapped in LiMnTiO_4_ particles. For superior performance aspects, such as flexibility, low weight, and high mechanical property, this material was used as a free-standing cathode and delivers a capacity of 161 mAh g^−1^ and 86.4% retention after 50 cycles at 0.5 C. This may be due to the promotion of Li^+^ and electron transformation through the porous, MWCNTs. More details about the comparison with some 3D Li-ion battery cathodes are listed in Table 1.

## 4. Methods for Constructing 3D Porous Li-S Battery Cathodes and Their Comparison

### 4.1. 3D Porous S Cathodes for Li-S Batteries

There are several reasons that the elemental sulfur itself cannot be fabricated as the cathode in Li-S batteries. One is that the sulfur is insulative and does not have a firm crystal to form a solid structure. In this case, after sulfur reacts with lithium ions, the resultant Li_2_S cannot be fixed in situ. The alternative method is to combine sulfur with other conductive materials. A frame strategy is promising and viable for solving the issues of elemental sulfur by coating sulfur onto a frame that has good conductivity, a large surface, and a porous structure [77,78,79]. Until now, carbon materials, conductive polymers, and metal or metal oxides have been studied for sulfur-based cathode materials. The energy stored in Li-S batteries is closely related to the mass of the sulfur. As such, 3D porous structures are desired to increase the mass loading.

Various kinds of carbon matrices, including carbon nanotubes, graphene, carbon nanofibers, carbon spheres, and mesoporous carbon, have been chosen to act as a frame for Li-S batteries. In addition, the fabrication of conceptually new structures is also attractive. For example, Lu et al. reported an approach to assemble a sulfur-graphene sponge with sulfur uniformly distributed into the pores of a graphene sponge [80]. The graphene sponge framework can not only improve the electronic conductivity but also accommodate the volume change in the charge and discharge process. The electrochemical measurement showed a high areal specific capacity of 4.53 mAh cm^−2^ after 300 cycles and a slow decay rate of 0.08% per cycle after 300 cycles at 0.1 C. To some extent, the interlaced graphene layer can absorb the polysulfides intermediate product. More performance comparison about 3D S cathodes are listed in Table 2.

Li et al. reported a novel 3D hierarchical polypyrrole loading sulfur [81]. Firstly, silica colloid crystal was used as a template to synthesize 3D polypyrrole, which possesses a large specific area. Subsequently, sulfur was loaded on the surface of polypyrrole via a solution-diffusion route followed by low-temperature treatment. When used as cathode for Li-S batteries, this material shows a high specific capacity of 751 mAh g^−1^ after 100 cycles at 0.1 C.

A wood-inspired multi-channel tubular graphene architecture prepared by chemical vapor deposition was also studied [82]. This unique structure provides a large interior space that accommodates sulfur and fixes the generated polysulfide. Meanwhile, the 3D multi-channel network also promotes mass transport and ion diffusion. The benefit from these properties is that the MCTG/S cathode for Li-S batteries delivers a discharge capacity of 1390 mAh g^−1^ at 0.1 C.

Two composites of ZnO@S/CNT and Ni(OH)_2_@S/CNT were reported and applied in Li-S batteries [83]. ZnO@S/CNT illustrates higher initial capacity of 1663 mAh g^−1^ and 56.6% retention after 70 cycles. It can be attributed to the strong interaction between ZnO and $S_{x}^{2-}$ based on density functional theory (DFT) calculation.

**Figure 9:**
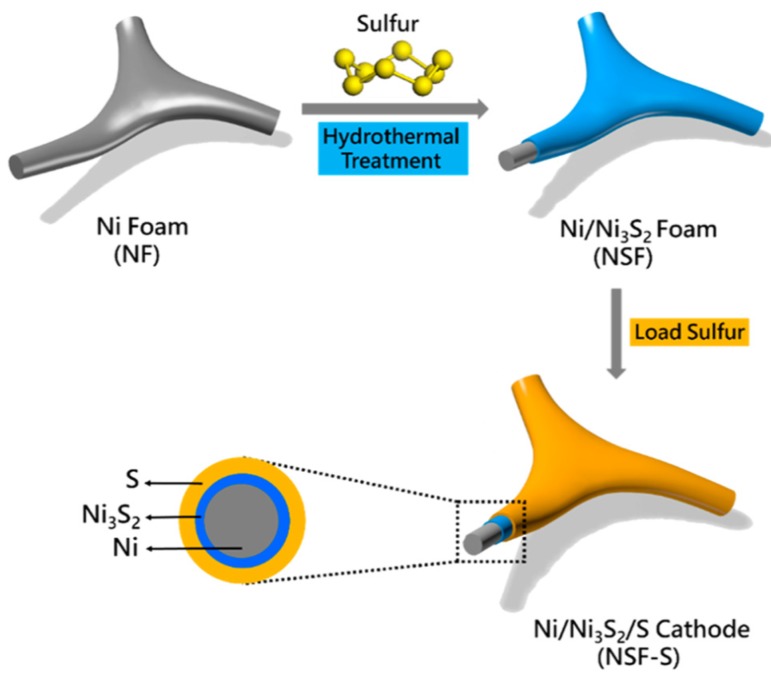
Schematic illustration of the Ni/Ni_3_S_2_/S hybrid cathode. Reproduced with permission from [84]. American Chemical Society, 2017.

**Figure 10:**
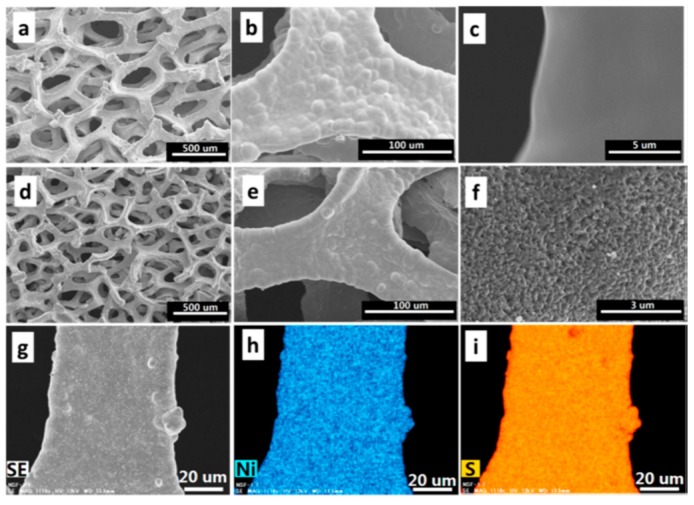
SEM images of (**a**–**c**) nickel foam and (**d**–**g**) nickel-sulfur foam at different magnifications. (**h**) Energy dispersive X-ray spectroscopy elemental mappings of nickel and (**i**) sulfur over image (**g**). Reproduced with permission from [84]. American Chemical Society, 2017.

Moreover, Li et al. synthesized Ni_3_S_2_ and coated it onto the surface of Ni foam by the hydrothermal method, followed by physically coating elemental S, and finally obtained 3D Ni/Ni_3_S_2_/S [84]. As displayed in Figure 9 and Figure 10, the reaction between Ni metal and sulfur generates Ni_3_S_2_ in situ on the surface of the Ni foam. Within the hybrid, the inner Ni metal network could serve as an electron transport pathway to facilitate the electrochemical reactions. In addition, the sufficient pore spaces enable a high sulfur loading and endure the volume change caused by the sulfur. When the loading amount is 4 mg cm^−2^, this material delivers a discharge capacity of 441 mAh g^−1^ after 100 cycles. 

**Figure 11:**
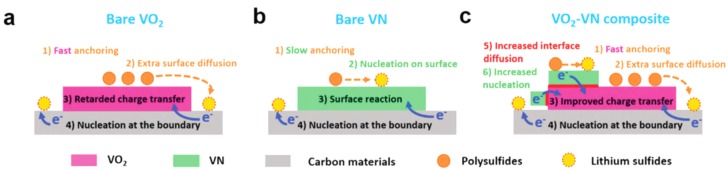
Illustration of LiPS anchoring-diffusion–conversion processes on VO_2_, VN, and VO_2_–VN binary host surfaces. Reproduced with permission from [85]. The Royal Society of Chemistry, 2018.

The in situ construction method was also used for preparing oxide-based composites. For example, Song et al. reported VO_2_-VN as an excellent sulfur host for Li-S batteries [85]. The S@VO_2_–VN cathode displayed a remarkable rate capacity of 935 mAh g^−1^ and 56.1% retention after 800 cycles at 2 C. As illustrated in Figure 11, the good performance ascribed to the improved interfacial contact between VO_2_ and VN built-up by the in situ construction route guarantees fast diffusion of LiPSs from VO_2_ to VN, resulting in an improved conversion and accelerated redox kinetics towards Li_2_S precipitation.

Several oxide-based cathodes were reported to possess the adsorptive capability towards polysulfides, including Li_2_S_2_, Li_2_S_4_, and Li_2_S_6_. It is necessary to reduce the shuttle effect of a Li-S battery, leading to improved stability. In contrast, the sulfur-based cathodes commonly suffer from loss of active materials because of the dissolving and transferring of polysulfides. The relatively high loading is more attractive for sulfur-based cathodes, as the oxide hosts are usually of large weight and volume compared to the active component sulfur within the electrodes. As for the preparation of Li-S battery cathode, many techniques used for making Li-ion battery cathodes are also applicable. Some methods are summarized in Table 2. However, what should be noted is that the low melting point of sulfur makes the high-temperature treatment unsuitable.

### 4.2. Brief Comparison between Li-Ion and Li-S Batteries 

As for the Li-ion batteries, they are advantageous as they show good safety, high working voltage, good specific capacity at low cycling rate, long cyclic performance, and low self-discharge. In parallel, for the Li-S batteries, capacities are commonly from 700 to 1500 mAh g^−1^, which are competitive with some other batteries. Nevertheless, there are some challenges for Li-S batteries, such as the dissolution of polysulfide in electrolyte, poor conductivity of sulfur, and the damage to the active materials caused from volume change. For example, the conductivity of S means it needs more conductive carbon and polymer binder in the cathode, which decreases the mass content of active materials within the electrode. Therefore, improving the capacity of the Li-ion battery and increasing the loading of sulfur in the Li-S battery are necessary to achieve a good energy density in practice.

## 5. Summary

Some important investigations on the 3D porous-structured electrodes for both Li-ion and Li-S batteries have been summarized. Compared to conventional thick-film electrodes, 3D porous electrodes enable fast electron and Li ion transfer, as well as a robust mechanical strength, and sufficient space for volume change accommodation during charge/discharge. However, 3D porous electrodes usually consume more electrolytes, which should be addressed in future studies. Potentially, constructing light-weight 3D frames and optimizing the porous structure could be promising. For such design and optimization, some artificial intelligence (AI) methods, such as genetic programming and automated neural network, are available [113]. They are able to establish models for optimizing battery pack enclosures for electric vehicles, and analyzing the capacities for electrodes [114,115], which is necessary for achieving an optimal 3D-structured electrode [116]. As demonstrative examples, LiCoO_2_, LiMn_2_O_4_, LiFePO_4_, V_2_O_5_, LiNi_1−x−y_Co_x_Mn_y_O_2_ in pristine and modified forms with a 3D porous structure for Li-ion batteries have been introduced, while S loaded on 3D nanoframes for Li-S batteries are discussed as well. The preparation approaches, including template-directed chemical vapor deposition, hydrothermal synthesis, atomic layer deposition, and the related electrochemical properties, are focused on. In view of the limited length of the mini review, some of the methods have not been discussed in depth. There are still some electrode materials out of the range of this review. However, we hope the review presented here could potentially be significant for researchers and engineers who are working in related fields. In addition, it is believed that 3D porous-structured materials for next generation cathodes are promising, which would be of benefit for developing high-performance advanced secondary batteries.

## Figures and Tables

**Table 1:** Comparison on the performance of three-dimensional Li-ion battery cathodes.

Cathode Materials	Preparation Method	Cycling Rate	Cycle Number	Capacity (mAh g^−1^)	References
Li_3_VP_3_ON	Solid-solid ion-exchange method	20 C	50	70	[51]
Li_3_V_2_(PO_4_)_3_/C	Sol-gel method	30 C	35	85	[36]
LiAlO_2_-LiMnPO_4_/C	Sol-gel method	10 C	100	105	[37]
(1 − x) LiNi_0.5_Mn_1.5_O_4_- xLi_2_SiO_3_	Sol-gel method	2 C	50	150.3	[52]
G/LiFePO_4_/G	Hydrothermal method	10 C	100	124	[53]
Li_3_Mo_4_P_5_O_24_	-	50 C	20	110	[54]
Li_2_FeSiO_4_/C	-	10 C	420	239	[55]
LiCaFeF_6_	Solid-state reaction	20 C	20	112	[41]
CNT@Li_2_MnSiO_4_@C	-	0.2 C	50	227	[56]
Li_3_FeF_6_	Sol-gel Mechanical stirring	50 mA g^−1^	100	174	[57]
LiFePO_4_@C	Hydrothermal	10 C	500	117	[58]
LiMnO_2_	In-situ carbothermal reduction method	0.1 C	40	165.3	[48]
LiCoPO_4_	Microwave-assisted solvothermal	0.1 C	20	141	[59]
Li_2_MnO_3_	Sol-gel method	0.1 C	100	225	[60]
Li_2_FeSiO_4_	Polyol method	20 C	50	270	[61]
LiVPO_4_F/C	Sol-gel method	10 C	20	121.1	[62]
LiMn_0.8_Fe_0.2_PO_4_	Solvothermal method	3 C	35	171	[63]
LiMnBO_3_@C	Sol-gel method	0.05 C	50	159.7	[64]
LiMn_2_O_4_	Hydrothermal method	0.2 C	1000	143.4	[65]
LiMnTiO_4_	Vacuum filtration method	0.5 C	50	161	[50]
Nano-SiO_2_@Li_2_CoPO_4_F	Hydrothermal method	2 C	60	79.4	[66]
LiVP_2_O_7_/C	Sol-gel method	0.05 C	50	102.3	[67]
LiFeBO_3_/C	Spray-drying	0.05 C	105	201.5	[68]
LiV_3_O_8_	High-temperature calcination	60 mA g^−1^	100	212.8	[69]
Li_2_CoSiO_4_/C	Hydrothermal method	36 mA g^−1^	100	144	[70]
LiMo_4_O_6_	Ion-exchange method	0.05 C	50	36.3	[71]
LiNi_0.5_Co_0.2_Mn_0.3_O_2_	-	0.1 C	100	164.6	[72]
LiCoBO_3_	Sol-gel method	10 C	52	98	[41]
LiNi_0.08_Mn_1.92_O_4_	Solution combustion method	1 C	1000	95.7	[73]
LiNi_0.6_Co_0.4-z_Ti_z_O_2_	Solid-state method	20	50	100	[42]
LiFe_0.4_Mn_0.4_Co_0.2_PO_4_/C	coprecipitation-and-milling method	1 C	100	104.7	[74]
Li_2_Ru_0.8_Ti_0.2_O_3_		100 mA g^−1^	90	196.1	[75]
Li_4_Ti_5_O_12_- LiNi_0.5_Mn_1.5_O_4_	Solvothermal method	0.5 C	100	122.6	[76]

**Table 2:** Comparison on the performance of 3D cathodes in Li-S batteries.

Cathode Materials	Preparation Method	Cycling Rate	Cycle Number	Capacity (mAh g^−1^)	S/E (Weight Ratio)	References
3D sulfur-doped graphene	One-pot wet chemical method	0.5 C	350	785	80%	[86]
Hierarchically porous nitrogen-doped carbon	-	0.1 C	300	1355	69%	[87]
α-MoO_3_	-	0.5 C	400	912.8	68%	[88]
Porous polypyrrole loading sulfur	Chemical polymerization method	0.1 C	100	751	59%	[81]
Wood inspired multi-channel tubular graphene	Template-directed chemical vapor deposition	0.1 C	500	1390	70%	[82]
3D N-doped graphene foam	Annealing method in chemical vapor deposition	0.2 C	200	819	2.05 mg cm^−2^	[89]
S/CeO_2_/RGO (reduced graphene oxide)	Hydrothermal method	0.1 C	20	792	64%	[90]
Li_2_S/graphene	Infiltration method	0.1 C	300	894.7		[91]
CNTs/MOFs-C/Al_2_(OH)_2.76_F_3.24_/S	-	500 mA g^−1^	300	889	72%	[92]
Polypyrrole@sulfur@polypyrrole	Chemical precipitation method	50 mA g^−1^	50	554	66%	[93]
Porous C_3_N_4_ nanosheets@RGO	-	0.5 C	800	680	68%	[94]
Titanium-dioxide-grafted carbon paper	-	0.5 C	200	850	40%	[95]
Nd_2_O_3_ nanoparticles doped carbon	Acid catalyzing method	0.2 C	100	1082	56%	[96]
CeF_3_-doped porous carbon nanofibers	Electroblown spinning technique and carbonization process	0.5 C	500	901.2	75%	[97]
NHCSs@MnO_2_/S	-	0.5 C	1000	1249	70%	[98]
ZnO@S/CNT	Ball-milling method	160 mA g^−1^	70	942	45%	[83]
TiN@CNT-S	-	0.05 C	80	1269	5.4 mg cm^−2^	[99]
NiCo_2_O_4_	Solvothermal method	0.2 C	200	1274	27%	[100]
S/AHCNS-SnS_2_	-	0.2 C	200	924	64%	[101]
Ni/Ni_3_S_2_/S	Hydrothermal method	4 mA cm^−1^	150	441	71%	[84]
TiB_2_/S	Melting-diffusion method	1 C	100	837	70%	[102]
Mn_3_O_4_@CNF/S		0.1 C	100	993	50%	[103]
Li_2_S/carbon	Icy water bathing method	0.2 C	150	595	-	[104]
Ti_4_O_7_ nanoparticle-embedded porous carbon	-	0.2 C	1000	1445	77%	[105]
S/Ti_3_C_2_T_x_	Melt-diffusion method	5 C	1500	608	80%	[106]
VO_2_-VN	Hydrothermal	1 C	800	1105	62%	[85]
Li_2_S@C-Co-N	Liquid infiltration-evaporation method	1 C	300	950.6	-	[107]
3D CNTs/Graphene-S-Al_3_Ni_2_	--	1 C	800	496	65%	[108]
Bamboo-like Co_3_O_4_	Hydrothermal method	1 C	300	796	72.6%	[109]
MnO_2_-Ti_3_C_2_	Electrostatic self-assembly approach	2 C	500	844.5	70%	[110]
S@TiO_2_/PPy	-	0.2 C	50	745.6	72.4%	[111]
TiC-TiO_2_/S	-	0.5 C	500	714	1.1 mg cm^−2^	[112]
